# Molecular detection and speciation of pathogenic *Leptospira *spp. in blood from patients with culture-negative leptospirosis

**DOI:** 10.1186/1471-2334-11-338

**Published:** 2011-12-13

**Authors:** Siriphan Boonsilp, Janjira Thaipadungpanit, Premjit Amornchai, Vanaporn Wuthiekanun, Wirongrong Chierakul, Direk Limmathurotsakul, Nicholas P Day, Sharon J Peacock

**Affiliations:** 1 Mahidol-Oxford Tropical Medicine Research Unit, Mahidol University, Bangkok, Thailand; 2 Medical Proteomics Unit, Office for Research and Development, Faculty of Medicine Siriraj Hospital, Bangkok, Thailand; 3 Department of Clinical Tropical Medicine,Faculty of Tropical Medicine, Mahidol University, Bangkok, Thailand; 4 Department of Tropical Hygiene, Faculty of Tropical Medicine, Mahidol University, Bangkok, Thailand; 5 Centre for Tropical Medicine, Nuffield Department of Clinical Medicine, University of Oxford, Churchill Hospital, Oxford, UK; 6 Department of Microbiology and Immunology, Faculty of Tropical Medicine, Mahidol University, Bangkok, Thailand; 7 Department of Medicine, University of Cambridge, Addenbrooke's Hospital, Cambridge, UK

## Abstract

**Background:**

Pathogenic *Leptospira *spp. present in the blood of patients with leptospirosis during the first week of symptoms can be detected using culture or PCR. A proportion of patients who are positive by PCR are negative by culture. *Leptospira *spp. are fastidious bacteria, and we hypothesized that a false-negative culture result may represent infection with a distinct bacterial subset that fail to grow in standard culture medium.

**Methods:**

We evaluated our hypothesis during a prospective study of 418 consecutive patients presenting to a hospital in northeast Thailand with an acute febrile illness. Admission blood samples were taken for *Leptospira *culture and PCR. A single tube nested PCR that amplified a region of the *rrs *gene was developed and applied, amplicons sequenced and a phylogenetic tree reconstructed.

**Results:**

39/418 (9%) patients were culture-positive for *Leptospira *spp., and 81/418 (19%) patients were culture-negative but *rrs *PCR-positive. The species associated with culture-positive leptospirosis (37 *L. interrogans *and 2 *L. borgpetersenii*) were comparable to those associated with culture-negative, PCR-positive leptospirosis (76 *L. interrogans*, 4 *L. borgpetersenii*, 1 unidentified, possibly new species).

**Conclusion:**

Molecular speciation failed to identify a unique bacterial subset in patients with culture-negative, PCR-positive leptospirosis. The rate of false-negative culture was high, and we speculate that antibiotic pre-treatment is the most likely explanation for this.

## Background

Leptospirosis is an acute febrile illness caused by pathogenic species belonging to the genus *Leptospira *[1,2]. This zoonotic disease has a worldwide distribution but is most common in tropical and subtropical regions and has the greatest impact on public health in developing countries [1-4]. Disease is maintained by chronic carrier hosts that excrete the organism into the environment, and infection in man results from direct contact with infected animals or indirect contact with a contaminated environment [1-3].

*Leptospira *are present in the blood during the first week of infective symptoms [1,2]. Culture is rarely performed in routine clinical practice since this may take several months and requires considerable expertise, which places it within the domain of specialist reference centres. Culture continues to have an important role, however, in defining the global epidemiology of infection [4]. Identification of the serovar of infecting isolates can provide clues as to the chronic carrier host, since certain serovars may by associated with one or a small number of mammalian or other species [2-4]. Such information makes an important contribution to the development of prevention strategies. Culture also provides vital material for basic and applied research [1,4].

Although culture provides valuable information and material, it is only positive in a minority of cases [5-8]. In a recent study conducted by us to assess the diagnostic accuracy of a real-time PCR assay for human leptospirosis in Thailand, culture was positive in only 39/133 (29%) laboratory confirmed cases [5]. The remaining cases were diagnosed using the microscopic agglutination test (MAT), a serologic test on paired (acute and convalescent) serum samples. MAT has been used previously to infer the prevalent *Leptospira *serovars in a given region by determining the serovar against which the highest titer is raised, but this is associated with considerable inaccuracy. In Thailand, MAT was reported to predict the infecting serovar in only 33% of human leptospirosis cases when compared with serovar determination of the actual infecting isolate using the cross agglutinin absorption test (CAAT) [9].

PCR is more sensitive than culture for the detection of *Leptospira *in clinical samples [5,8,10,11]. In our setting, PCR detects around twice as many cases of leptospiraemia compared with culture in patients presenting to hospital with an acute febrile illness [5]. *Leptospira *spp. are fastidious microorganisms, and a possible explanation is that patients who are culture-negative but PCR-positive are infected with isolates that fail to grow in the standard culture medium used (EMJH, Ellinghausen, McCullough, Johnson, and Harris medium). Here, we explore this possibility by examining the hypothesis that *Leptospira *spp. associated with culture-negative leptospirosis includes a distinct subset at the species level compared with *Leptospira *spp. associated with culture-positive infection.

## Methods

### Ethics Statement

Ethical approval was given by the Ministry of Public Health, Royal Government of Thailand for the cohort study of acute febrile illness conducted in northeast Thailand from which the subjects and blood samples used here were drawn. These patients provided written informed consent [5]. Additional ethical approval was given by the Faculty of Tropical Medicine, Mahidol University, Thailand for their inclusion in this case-control study.

### Patient recruitment, sampling and *Leptospira *culture

A prospective cohort of 418 consecutive patients presenting to the Udon Thani hospital, northeast Thailand with an acute febrile illness were recruited between 10^th ^January 2001 and 16^th ^June 2002, as described previously [5]. In brief, patients were identified during twice daily rounds of the medical wards. Inclusion criteria were patients who were ≥ 15 years of age with fever (> 37.8°C) of unknown cause who agreed to participate in the study. Patients with a blood smear positive for malaria parasites or those with another definable source of infection on admission such as pneumonia or urinary tract infection were excluded from the study. Parallel blood samples for culture and PCR (5 ml collected into each of heparin and EDTA blood tubes, respectively) were taken on admission from all patients. *Leptospira *culture was established in Udon Thani hospital on the day of venepuncture, and cultures were maintained and examined weekly for up to 6 months, as described previously [12]. Species identification of *Leptospira *cultures was undertaken by amplification and sequencing of the near full-length 16S rRNA gene, as described previously [6]. EDTA samples were left at room temperature for no more than 6 hours prior to storage at -80°C.

### Bacterial strains and DNA extraction

The *Leptospira *spp. used in this study are listed in Table 1. Additional isolates used were one clinical isolate of each of the following: *Staphylococcus aureus*, *Enterococcus *spp., *Escherichia coli*, *Salmonella enterica *serovar Typhi, *Klebsiella pneumoniae*, *Pseudomonas aeruginosa*, *Burkholderia pseudomallei*, *Orientia tsutsugamushi *strain Kato and *Rickettsia typhi*. Genomic DNA was extracted from laboratory cultures using the Wizard^® ^Genomic DNA extraction kit (Promega, USA), with the addition of 5 μl of 10 mg/ml lysostaphin during the extraction of *S. aureus*. The EDTA blood sample was thawed and total DNA extracted in 2009. Extraction was performed using the Nucleon™ BACC Genomic DNA Extraction Kit (GE Healthcare Biosciences, USA) and the extract suspended in 1 ml of TE buffer. DNA samples were stored at -20°C prior to use.

**Table 1:** *Leptospira *spp.used in this study

Serovar	Serogroup	Strain	Species	Status
Lai	Icterohaemorrhagiae	Lai	*L. interrogans*	Pathogen

Cynopteri	Cynopteri	3522C	*L. kirschneri*	Pathogen

Fortbragg	Autumnalis	Fort Bragg	*L. noguchii*	Pathogen

Javanica	Javanica	Veldrat Batavia 46	*L. borgpetersenii*	Pathogen

Manhao3	Manhao	L60	*L. alexanderi*	Pathogen

Alice	Autumnalis	Alice	*L. santarosai*	Pathogen

Pingchang	Ranarum	80-412	*L. alstonii*	Pathogen

Sarmin	Sarmin	Sarmin	*L. weilii*	Pathogen

Lyme	Lyme	10	*L. inadai*	Intermediate

Khorat	-	H2	*L. wolffii*	Intermediate

Hualin	Icterohaemorrhagiae	LT11-33	*L. terpstrae*	Non-pathogen

Semaranga	Semaranga	Veldrat Semaranga 173	*L. meyeri*	Non-pathogen

Patoc	Semaranga	Patoc I	*L. biflexa*	Non-pathogen

Codice	Codice	CDC	*L. wolbachii*	Non-pathogen

Saopaulo	Semaranga	Sao Paulo	*L. yanagawae*	Non-pathogen

### Primer design

Primer design was based on three criteria. These were that primers would: (i) anneal to DNA belonging to *Leptospira *spp. associated with disease in humans or animals (i.e. belonging to the pathogenic and intermediate groups), but not to saprophytes which can be laboratory contaminants; (ii) amplify a gene fragment small enough to be sequenced in a single reaction; and (iii) amplify a gene fragment containing the maximal number of polymorphic sites between species for the size of product, maximizing the ability of the region to discriminate between different but closely related *Leptospira *species.

We downloaded and aligned 184 sequence traces from GenBank for the partial or full *rrs *gene of *Leptospira *spp. belonging to nine pathogenic and five intermediate species (see Additional file, Table S1 for accession numbers). The number for each species was as follows - pathogenic species: *L. interrogans *(n = 61), *L. borgpetersenii *(n = 28), *L. kirschneri *(n = 21), *L. santarosai *(n = 22), *L. noguchii *(n = 12), *L. weilii *(n = 13), *L. alexanderi *(n = 5), *L. alstonii *(n = 2), *L. kmetyi *(n = 1); intermediate species: *L. broomii *(n = 3), *L. fainei *(n = 3), *L. inadai *(n = 3), *L. licerasiae *(n = 9), and *L. wolffii *(n = 1). The median length of sequence traces was 1320 nucleotides (range 977-1509, IQR = 1318-1431); the 1509 nucleotide trace represents the full-length *rrs *of *L. interrogans *serovar Lai. A 443-nucleotide region was identified that contained 32% (45/141) of all polymorphic sites within the 184 sequence trace alignment. This was located at positions 89 to 531 of the *rrs *gene of *L. interrogans *serovar Lai strain 56601 [GenBank: NC_004342].

### Single tube nested PCR assay

Molecular assays were performed at the Mahidol-Oxford Tropical Medicine Research Unit. A single tube nested PCR was developed that would amplify the 443-nucleotide *rrs *fragment described above, together with around 50 bases up- and downstream of this target region. PCR primers were designed using PrimerSelect software (DNASTAR Inc., Wisconsin, USA). Primers were as follows: rrs-outer-F (5' CTCAGAACTAACGCTGGCGGCGCG 3'), rrs-outer-R (5' GGTTCGTTACTGAGGGTTAAAACCCCC3'), rrs-inner-F (5' CTGGCGGCGCGTCTTA 3'), and rrs-inner-R (5' GTTTTCACACCTGACTTACA 3'). The resulting amplicon was 547 bp. A 25 μl PCR reaction contained 4.5 mM of MgCl_2_, 200 μM of dNTP, 1.25 unit of Tag DNA polymerase (Roche, USA), 0.150 pmol of each outer primer, 1.25 pmol of rrs-inner-F, 5 pmol of rrs-inner-R, 1 M of Betaine (Sigma-Aldrich, USA) and either 1 μl of DNA extracted from laboratory cultures or 5 μl of DNA extracted from EDTA blood samples taken from febrile patients. PCR was performed in duplicate for each sample using a PTC-200 Peltier Thermal Cycler (MJ research, USA) using the following conditions: one cycle of 95°C for 2 minutes; 40 cycles of 95°C for 10 seconds, 67°C for 15 seconds and 72°C for 30 seconds; 40 cycles of 95°C for 10 seconds, 55°C for 15 seconds and 72°C for 30 seconds; then one cycle of 72°C for 7 minutes. Positive and negative controls were included in each run. The positive control was genomic DNA extracted from *L. interrogans *serovar Lai strain Lai spiked into human DNA as diluent. Human DNA was extracted from a 5 ml blood sample taken from a single individual and extracted as described above for clinical samples. The negative control was reaction mixture minus DNA template. Amplicons were visualized using 1.5% gel electrophoresis followed by staining with ethidium bromide. A positive PCR result was defined as the visualization of a band of predicted size for one or both samples.

Analytical sensitivity of the assay was determined using 10-fold serial dilutions of genomic DNA extracted from *L. interrogans *serovar Lai strain Lai and suspended in human DNA as diluent. Genomic equivalents (GE) were estimated based on a genomic size of 4,659,275 bp, the average genome size of two *L. interrogans *strains [13,14]. Analytical specificity was determined using DNA extracted from *Leptospira *spp. (Table 1) and other pathogenic bacteria that are common causes of septicemia or febrile illness in our setting (*S. aureus*, *Enterococcus *spp., *E. coli*, *S. enterica *serovar Typhi, *K. pneumoniae*, *P. aeruginosa*, *B. pseudomallei*, *O. tsutsugamushi *and *R. typhi*).

### Sequencing and sequence analysis

Purified PCR products were sequenced by Macrogen Inc. (Seoul, Korea) using the rrs-inner-F and rrs-inner-R primers. Sequences were aligned using SeqManII software (DNASTAR Inc., Wisconsin, USA), and trimmed to the 443-nucleotide region. A neighbor-joining tree was reconstructed prior to inferring *Leptospira *spp. based on a comparison with sequence for 14 pathogenic and intermediate *Leptospira *spp. acquired from GenBank database using software Mega 5.0 [15].

## Results

### Discriminatory power of the 443-nucleotide region to speciate *Leptospira *spp

**Figure 1:**
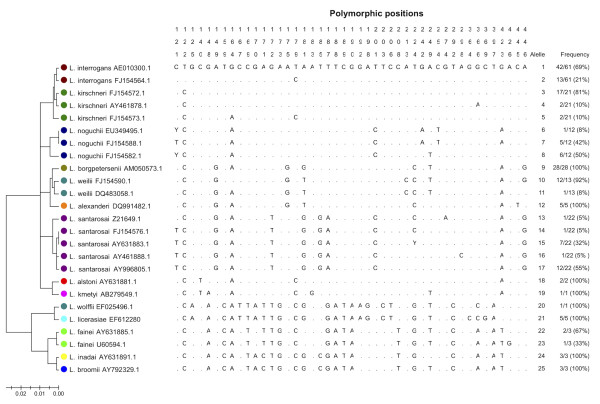
**Phylogenetic analyses of 184 partial *rrs *sequences from 14 *Leptospira *species resolves 25 unique alleles**. The tree was reconstructed using the neighbor-joining method as implemented in MEGA ver 5.0 [15]. A total of 45 polymorphic sites were demonstrated. The vertical numbers shown indicate the nucleotide position based on *rrs *of *L. interrogans *serovar Lai [GenBank: AE01300.1], and a dot indicates an identical nucleotide at that position.

Alignment of the 443-nucleotide *rrs *fragment of 184 strains revealed a total of 25 unique alleles for 14 different species. Polymorphic sites, a phylogenetic tree and the frequency of each allele by species are shown in Figure 1. Eight *Leptospira *spp. (*L. borgpetersenii*, *L. alexanderi*, *L. alstonii*, *L. kmetyi*, *L. wolffii*, *L. inadai*, *L. licerasiae*, and *L. broomii*) each had a single unique *rrs *type (of which two species, *L. kmetyi *and *L. wolffii*, only had one *rrs *sequence trace available for the analysis). The remaining 6 species had between two to five alleles; the frequency of each allele within a given species is shown in Figure 1.

An analysis was performed to determine the discriminatory power of the 443-nucleotide *rrs *fragment to speciate the 14 *Leptospira *spp. compared with the nearly whole length *rrs *gene (median length 1320 bp) for the 184 sequence traces by comparing average (%) nucleotide distances. Average distances ranged from 0.27-2.50% between the pathogenic species and 0.23-3.16% between the intermediate species for the 443-nucleotide region (Table 2). By comparison, average distances ranged from 0.13-1.42% between pathogenic species and 0.23-2.73% between the intermediate species for the near full-length gene (Table 2). This indicates that the use of the 443-nucleotide *rrs *fragment is associated with a similar discriminatory power to that of longer regions of the *rrs *gene sequence.

**Table 2:** Nucleotide distance based on the 443-nucleotide fragment and almost full-length fragment of the *rrs *gene

Nucleotide distance based on the 443-nucleotide *rrs*													
**Average distance (%)**													

	**1**	**2**	**3**	**4**	**5**	**6**	**7**	**8**	**9**	**10**	**11**	**12**	**13**

1 *L. interrogans*													

2 *L. kirschneri*	0.27												

3 *L. borgpetesenii*	1.81	1.58											

4 *L. noguchii*	1.13	0.90	1.58										

5 *L. santarosai*	2.28	2.05	1.37	1.83									

6 *L. weilii*	1.81	1.62	0.68	2.00	2.05								

7 *L. alexanderi*	1.58	1.35	0.90	1.58	1.83	1.09							

8 *L. alstonii*	0.90	0.68	1.35	0.90	1.83	1.77	1.13						

9 *L. kmetyi*	1.58	1.35	1.58	1.35	2.50	2.00	1.81	0.68					

10 *L. fainei*	4.14	3.87	4.36	4.03	4.61	4.78	4.59	3.91	3.69				

11 *L. inadai*	4.51	4.25	4.74	4.63	4.31	5.38	4.74	4.29	4.51	1.20			

12 *L. broomii*	4.74	4.47	4.51	4.63	4.54	5.16	4.97	4.51	4.29	0.98	0.23		

13 *L. wolffii*	5.42	5.17	5.42	5.42	5.66	6.06	5.87	5.42	5.19	2.56	2.71	2.48	

14 *L. licerasiae*	5.87	5.62	5.87	5.87	6.12	6.51	6.32	5.87	5.64	3.01	3.16	2.93	0.45

**Nucleotide distance based on almost whole length fragment of *rrs *gene**													

**Average distance (%)**													

	**1**	**2**	**3**	**4**	**5**	**6**	**7**	**8**	**9**	**10**	**11**	**12**	**13**

1 *L. interrogans*													

2 *L. kirschneri*	0.13												

3 *L. borgpetesenii*	0.77	0.69											

4 *L. noguchii*	0.64	0.57	0.83										

5 *L. santarosai*	1.11	1.03	0.81	0.91									

6 *L. weilii*	1.00	0.92	0.48	1.00	1.05								

7 *L. alexanderi*	0.99	0.89	0.63	1.06	1.23	0.92							

8 *L. alstonii*	0.89	0.83	1.08	0.81	1.42	1.28	1.24						

9 *L. kmetyi*	0.95	0.89	0.99	0.80	1.35	1.21	1.27	0.53					

10 *L. fainei*	4.93	4.81	4.86	4.86	5.09	5.22	5.24	5.11	4.62				

11 *L. inadai*	4.84	4.71	4.79	4.90	4.86	5.27	5.09	5.04	4.77	0.61			

12 *L. broomii*	5.06	4.94	4.86	5.01	5.01	5.27	5.32	5.27	4.77	0.43	0.23		

13 *L. wolffii*	4.62	4.54	4.58	4.72	4.80	4.98	4.86	4.85	4.55	2.42	2.58	2.35	

14 *L. licerasiae*	4.32	4.24	4.30	4.27	4.42	4.58	4.48	4.47	4.09	2.65	2.73	2.58	1.36

### Analytical sensitivity and specificity of single tube nested PCR

The analytical sensitivity of the PCR assay was determined using DNA of *L. interrogans *serovar Lai strain Lai diluted to reach a final concentration of 100, 10 or 1 GE/reaction. Duplicate samples at each DNA concentration were evaluated on 10 independent occasions. Both samples were positive on all occasions for 100 and 10 GE/reaction, while 8/10 runs were positive for 1 GE/reaction (both samples positive in 6/10 runs and one of two samples positive in 2/10 runs). An LOD value of 1 GE/reaction equates to 40 *Leptospira *cells per 1 ml of human blood using the DNA extraction and PCR protocol described in materials and methods. Analytical specificity was determined using DNA from 15 *Leptospira *strains belonging to pathogenic, intermediate or non-pathogenic *Leptospira *spp. (Table 1), together with 9 other bacterial species that frequently cause febrile illness in our population. The assay gave a positive reaction for all *Leptospira *spp. in the pathogenic and intermediate groups, and was negative for *Leptospira *spp. belonging to the non-pathogenic group. All reactions were negative using DNA from one representative each of *S. aureus, Enterococcus *sp., *E. coli, S. enterica *serovar Typhi, *K. pneumoniae, P. aeruginosa, B. pseudomallei, O. tsutsugamushi *and *R. typhi*.

### *Leptospira *spp. associated with culture-positive and culture-negative leptospirosis

Admission blood samples were taken for *Leptospira *culture and PCR from 418 consecutive patients presenting to a hospital in northeast Thailand with an acute febrile illness. Culture was positive in 39/418 cases (9%); the infecting species in these cases (determined by extraction of genomic DNA from cultured organisms, amplification and sequencing of the near full-length *rrs *gene) were *L. interrogans *(n = 37) and *L. borgpetersenii *(n = 2).

**Figure 2:**
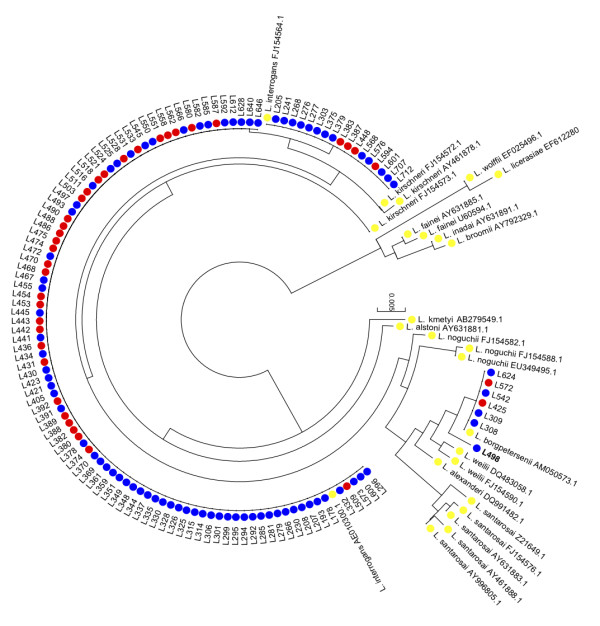
**Phylogenetic analysis of a 443-nucleotide region of *rrs *for 118 isolates of 14 *Leptospira *species**. The tree was reconstructed using the neighbor-joining method as implemented in MEGA ver 5.0 [15]. Colour coding represents the following: RED, sequence of a PCR fragment amplified from patients with culture-positive leptospirosis; BLUE, sequence of a PCR fragment amplified from patients with culture-negative leptospirosis; YELLOW, sequence for reference strains obtained from GenBank.

The 443-nucleotide *rrs *PCR assay was performed on all 418 samples. This was positive in 37/39 of the culture positive cases. A comparison of the sequence from the two PCR assays demonstrated that the 443-nucleotide *rrs *fragment amplified directly from blood was identical to the same region of the near-full length *rrs *gene amplified from cultured organisms in each case. The 443 bp *rrs *PCR was also positive for 81 of the 379 culture negative cases. The infecting species in these cases were defined based on phylogenetic analysis of the *rrs *fragment as *L. interrogans *(n = 76), *L. borgpetersenii *(n = 4), and an unidentified species (n = 1, sample L498) (Figure 2). All 118 sequences of the 443-nucleotide *rrs *have been submitted to GenBank [GenBank: JF925163 - JF925280].

### Analysis of an unidentified *Leptospira *species

**Figure 3:**
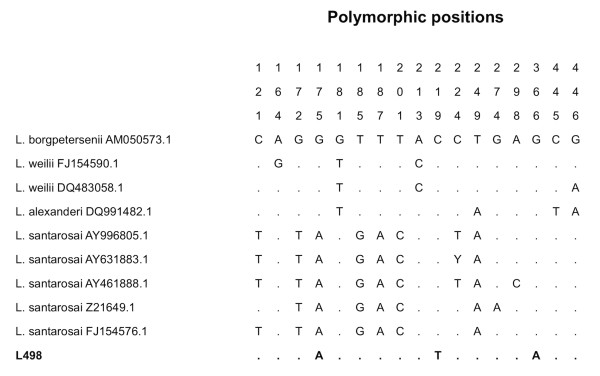
**Comparison of polymorphic sites in a 443-nucleotide region of *rrs *for an unidentified *Leptospira *species**. The sequence of L498 was compared with that of four species (nine different *rrs *alleles) with which it genetically clusters.

The *rrs *sequence of the unidentified species from sample L498 clustered in a phylogenetic branch of the tree containing *L. borgpertersenii*, *L. santarosai*, *L. weilii *and *L. alexanderi*, and was most closely related to *L. borgpetersenii *(Figure 2). The 443-nucleotide *rrs *sequence of L498 was aligned with the nine *rrs *alleles identified for these four species (Figure 3). This demonstrated a total of three polymorphic sites for the unidentified species, as follows: A at position 175, T at position 219, and A at position 366. The presence of T at position 219 was unique in the entire *rrs *database for all 14 species, while the presence of an A at position 175 was observed in multiple species (Figure 1). The presence of an A at position 366 was observed in one of the three *rrs *alleles of *L. kirschneri*, and in three intermediate *Leptospira *species (*L. fainei*, *L. inadai *and *L. broomii*) (Figure 1).

The patient from whom this sample was collected presented with 3 days of fever, headache, conjunctival suffusion and calf tenderness. Paired serum samples were taken on days 2 and 10 after the onset of symptoms from this patient. MAT was negative (MAT titer < 1:50) against a panel of reference isolates of the following serovar: Pomona, Hardjo, Tarassovi, Grippotyphosa, Celledoni, Copenhageni, Australis, Pyrogenes, Canicola, Hebdomadis, Mini, Sarmin, Autmnalis, Cynopteri, Ballum, Bataviae, Djasiman, Javanica, Panama, Shermani and Mwalok. The patient became afebrile eight hours after the initiation of doxycycline, made an uneventful recovery, and was discharged home three days after admission. Doxycycline was continued to complete a 7-day course. The patient had made a full clinical recovery when seen in clinic for the day 10 serum sample.

## Discussion

The purpose of this study was to examine whether *Leptospira *spp. associated with culture-negative leptospirosis included a distinct subset at the species level compared with *Leptospira *spp. associated with culture-positive infection. The driver for this was to explain why the majority of patients with PCR-positive leptospirosis in our setting are culture-negative. Demonstration of a specific culture-negative sub-set would have opened the way to develop alternative culture media and potentially increase the sensitivity of culture, but we found no such differences.

There are several alternative explanations for PCR-positive but culture-negative leptospirosis. The patient may have received an effective antimicrobial drug but may not have cleared non-viable organism by the time that the blood sample was taken. We do not have information on antimicrobial consumption by our study patients prior to hospital admission, and this remains an important possibility. *Leptospira *spp. might also perish in the blood collection tube prior to laboratory culture due to fluctuations in ambient temperature or other factors, and are fastidious bacteria with highly defined growth requirements. Timing of the sample after the onset of symptoms is also important; we have reported previously for the same group of patients that those who were PCR-positive and culture-positive had a shorter duration of illness prior to sampling than those who were PCR-positive but culture-negative [6]. Two patients were culture-positive but PCR-negative. A possible explanation is that this resulted from a stochastic effect associated with a sample containing a low bacterial concentration; i.e., by chance, the aliquot taken from the sample for culture contained organisms but the sample used for DNA extraction did not. An alternative explanation is laboratory error or contamination.

This study has several limitations. The patient group studied were those people who were sufficiently unwell to require hospitalization, and so the findings cannot be generalized to cases with milder clinical manifestations. Furthermore, the characteristics of *Leptospira *spp. and the study population in Thailand may be significantly different to that in other settings.

The genetic diversity of *rrs *described here has been noted previously during a study in which a 452-nucleotide region of the 16S ribosomal RNA gene (defined with reference to *rrs2 *of *L. interrogans *serovar Copenhageni strain Fiocruz L1-130) was used as one of six loci of a genotyping scheme devised by Ahmed et al. [10]. This region maps to positions 103 to 554 of the *rrs *gene of *L. interrogans *serovar Lai strain 56601, and includes the 443-nucleotide *rrs *region used in our study (position 89 to 531). The number of alleles and polymorphic sites in 9 pathogenic and 5 intermediate *Leptospira *spp. were identical for the 443-nucleotide and 452-nucleotide regions. Ahmed et al. evaluated 120 strains belonging to 6 pathogenic species (*L. interrogans*, *L. noguchii*, *L. kirschneri*, *L. santarosai*, *L. alexanderi*, and *L. borgpetersenii*), which were separated by *rrs*2 into 29 different alleles containing 20 polymorphic sites. This suggests that alleles of *Leptospira rrs *arise frequently and could occur through homologous recombination within or between closely related species rather than by mutation [16,17].

Gene sequence analysis represents an important tool for the delineation of *Leptospira *species. The genes used previously in such analyses have included 16S rRNA (*rrs*) [18-29], DNA gyrase subunit B (*gyrB*) [26,30-32] and RNA polymerase subunit B (*rpoB*) [33,34]. An important question is what constitutes a new *Leptospira *species based on genetic differences at one or a small number of genetic loci. At one end of the spectrum, a *Leptospira *species may be clearly genetically distinct from other members of the genus based on a large degree of genetic diversity. Nucleotide distances between pathogenic and intermediate species evaluated in this study were more that 4% when compared using the nearly entire *rrs *gene (> 3% based on the 443-nucleotide *rrs *region). However, defining a species based on genetic differences at a small number of genetic loci is problematic. For example, two closely related *Leptospira *species (*L. interrogans *and *L. kirschneri*) have just 1 to 4 nucleotide differences when compared using the nearly entire gene, and 1 to 3 nucleotide differences based on the 443-nucleotide *rrs *region. Similarly, the sequence difference between the closely related *L. inadai *and *L. broomii *is 3 nucleotides based on the nearly entire gene and a single nucleotide based on the 443-nucleotide *rrs *region. This is equivalent to the number of differences observed within *L. noguchii *alone, which using our 443-nucleotide *rrs *region defined 5 polymorphic sites and 3 alleles. The unidentified species in this study (L498) was most closely related to *L. borgpetersenii *and had 3 nucleotide differences based on the 443-nucleotide *rrs *region. It remains to be seen whether this is a new species or a variant of an existing one. The lack of a live bacterial culture in this case precludes further characterization using additional tools such as DNA hybridization.

## Conclusions

The PCR assay described here was designed to speciate *Leptospira *spp. associated with culture-negative infection, and increases the number of cases for whom the causative species can be identified. This assay also represents a potentially useful epidemiological tool in settings where culture is not available. It is not clear why culture-negative leptospirosis is so common among our patient population, but the ready availability of over the counter antibiotics and the frequency with which these are purchased represents a plausible explanation that could be tested in future studies.

## Competing interests

The authors declare that they have no competing interests.

## Authors' contributions

JT, SP, SB, VW, WC and ND conceived and designed the study. SB and PA performed the laboratory work, SB, JT and DL analyzed the data and JT, SP and SB wrote the manuscript. All authors read and approved the final manuscript.

## Pre-publication history

The pre-publication history for this paper can be accessed here:

http://www.biomedcentral.com/1471-2334/11/338/prepub

## Supplementary Material

Additional file 1 **Additional Table Accession numbers of *rrs *sequences used during primer design**. Click here for file
