# Supplementary material for: Molecular detection and speciation of pathogenic Leptospira spp. in blood from patients with culture-negative leptospirosis
Source: BMC Infect Dis. 2011 Dec 13;11:338. doi: 10.1186/1471-2334-11-338 (PMC3297668; doi:10.1186/1471-2334-11-338)
Supplement: Additional file 1 — Additional Table Accession numbers of rrs sequences used during primer design. [file 1471-2334-11-338-S1.DOC]

**Additional Table 1. Accession numbers of *rrs* sequences used during primer design**

| **Species** | **GenBank ID of *rrs* sequences** |
| --- | --- |
| *L. interrogans* | AE010300.1, FJ154569.1, FJ154566.1, FJ154564.1, FJ154563.1, FJ154561.1, FJ154558.1, FJ154557.1, FJ154556.1, FJ154553.1, FJ154552.1, FJ154551.1, FJ154550.1, FJ154549.1, FJ154547.1, FJ154545.1, FJ154544.1, FJ154543.1, FJ154542.1, DQ991474.1, DQ991473.1, DQ991472.1, DQ991471.1, DQ991470.1, DQ991469.1, DQ991468.1, DQ991467.1, DQ991466.1, DQ991465.1, DQ991464.1, AY461870.1, AY461871.1, AY461866.1, AY461868.1, AY461865.1, AY461864.1, AY996790.2, AY461863.1, AY996800.1, AM050586.1, AM050585.1, AM050584.1, AM050583.1, AM050582.1, AM050580.1, AM050579.1, AM050578.1, AM050571.1, AM050570.1, AM050568.1, AM050566.1, AM050565.1, AY996798.1, AY996797.1, AY996796.1, AY996794.1, AY996793.1, AY996792.1, AY631894.1, EF596782.1, EU581713.1 |
| *L. kirschneri* | FJ154572.1, FJ154573.1, FJ154560.2, AY461879.1, AY461878.1, AY461877.1, AY996801.1, AM050567.1, AY996802.1, AY631895.1, AY461874.1, AY461873.1, DQ991479.1, DQ991478.1, DQ991477.1, DQ991476.1, DQ991475.1, FJ154562.1, FJ154559.1, AM050574.1, FJ154546.1 |
| *L. borgpetersenii* | AM050573.1, FJ154596.1, EF596783.1, EF467916.1, FJ154600.1, FJ154595.1, FJ154593.1, FJ154586.1, FJ154592.1, DQ991485.1, AY461862.1, AY461861.1, AY461860.1, AY461859.1, AY461858.1, AY461857.1, DQ991484.1, DQ991483.1, AM050569.1, AM050577.1, AM050581.1, AM050576.1, AM050572.1, FJ154591.1, AY461856.1, AY461855.1, AY631884.1, AY887899.1 |
| *L. noguchii* | FJ154588.1, FJ154574.1, EU349495.1, FJ154582.1, EU349496.1, DQ991500.1, DQ991499.1, DQ991498.1, DQ991497.1, AY461881.1, AY461880.1, AY631886.1 |
| *L. santarosai* | AY996805.1, AY631883.1, FJ154598.1, FJ154589.1, FJ154585.1, AY461890.1, AY461889.1, AY461888.1, AY461887.1, DQ991496.1, DQ991495.1, DQ991494.1, DQ991493.1, Z21649.1, DQ991492.1, DQ991491.1, AY461884.1, AY461885.1, AY461886.1, FJ154584.1, FJ154583.1, FJ154576.1 |
| *L. weilii* | FJ154590.1, DQ483058.1, FJ154581.1, DQ991490.1, FJ154580.1, DQ991489.1, DQ991488.1, AY631877.1, AY034037.1, AY461892.1, DQ991487.1, DQ991486.1, Z21637.1 |
| *L. alexanderi* | DQ991482.1, DQ991481.1, AY996804.1, AY996803.1, AY631880.1 |
| *L. kmetyi* | AB279549.1 |
| *L. licerasiae* | EF612280, EF612281, EF612282, EF612283, EF612284, EF612285, EF612286, EF612287, EF612288 |
| *L. fainei* | AY996789.1, AY631885.1, U60594.1 |
| *L. inadai* | AY631891.1, AY631896.1, AY631887.1 |
| *L. wolffii* | EF025496.1 |
| *L. broomii* | AY792329.1, AY796065.1, Y19243.1 |
| *L. alstonii* | AY631881.1, DQ991480.1 |
